# A Multicentre Study of Burnout Prevalence and Related Psychological Variables in Medical Area Hospital Nurses

**DOI:** 10.3390/jcm8010092

**Published:** 2019-01-15

**Authors:** Lucia Ramirez-Baena, Elena Ortega-Campos, Jose Luis Gomez-Urquiza, Gustavo R. Cañadas-De la Fuente, Emilia I. De la Fuente-Solana, Guillermo A. Cañadas-De la Fuente

**Affiliations:** 1 Brain, Mind and Behaviour Research Center (CIMCYC), University of Granada, Campus Universitario de Cartuja S.N., 18011 Granada, Spain; luciarb@correo.ugr.es (L.R.-B.); edfuente@ugr.es (E.I.D.l.F.-S.); 2 Faculty of Psychology, University of Almería, Carretera Sacramento S.N., 04120 Almería, Spain; elenaortega@ual.es; 3 Faculty of Health Sciences, University of Granada, Avenida de la Ilustración N. 60, 18016 Granada, Spain; gacf@ugr.es; 4 Faculty of Educational Sciences, University of Granada, Campus Universitario de Cartuja S.N., 18011 Granada, Spain; grcanadas@ugr.es

**Keywords:** burnout, medical area, medical nursing, nursing, personality factors, risk factors

## Abstract

Background: Nursing burnout is an important problem that affects nurses’ wellness, the quality of care and the health institutions. Study aims were to estimate levels of burnout; to determine the phase of burnout experienced by nurses in the medical area; to analyse the relationship between burnout and personality and psychological factors. Methods: Quantitative, cross-sectional, multicentre study. Hospitals from eight cities were included. The study sample was *n* = 301 nurses, working in the medical area of hospitals in the Andalusian Health Service during the second semester of 2017. Sociodemographic, occupational and personality variables were studied using the Revised NEO Personality Inventory together with the Educational-Clinical Questionnaire: Anxiety and Depression, and burnout was measured by the Maslach Burnout Inventory. Results: Almost 40% of the nurses presented high levels of burnout. The three burnouts (emotional exhaustion, depersonalisation and personal accomplishment) presented statistically significant correlations with the personality factors of neuroticism, extraversion, openness, agreeableness and conscientiousness, and also with the scores recorded for anxiety and depression. Multiple linear regression models showed agreeableness and depression to be statistically significant predictors of all dimensions of the syndrome. Conclusion: Hospital nurses working in the medical area in Andalusia experience high levels of burnout.

## 1. Introduction

Burnout syndrome appears in response to chronic work stress, provoking a crisis in the relationship with work itself [1,2]. These referenced authors presented burnout as a three-dimensional syndrome characterised by: (1) emotional exhaustion (EE), or feelings of physical over-exertion and emotional fatigue as a result of the continuous interactions required between the worker and users of his/her services; (2) depersonalisation (D), or the existence of cynical attitudes and responses towards the persons to whom services are provided; and (3) the sensation of little personal accomplishment (PA), i.e., the loss of confidence and the appearance of a negative self-concept due to encounters with unrewarding situations. 

In the present study, burnout was measured using the Maslach Burnout Inventory (MBI) [2], a questionnaire based on EE, D and PA and which is now the most widely-used instrument for this purpose [3,4]. The scores obtained for each of the MBI dimensions were categorised as ‘high’ or ‘low’, from which eight phases of progressive burnout were defined. On this basis, we propose explanatory models that enable us to assess the development of the disorder, as previously shown in the Golembiewski model [5].

Since 2010, the International Labor Organization has considered burnout syndrome to be an occupational disease, and the WHO has termed it the epidemic of the 21st century [6], affecting a broad spectrum of workers [7]. Professional practice demanding considerable emotional involvement makes the syndrome more likely to develop [8] and therefore healthcare workers, and nurses in particular, are among the major risk groups [9]. Studies have reported high values in this respect for nurses [10,11], although no exact figure has yet been offered. This situation should be taken very seriously, as the consequences for workers’ health, for institutions and for patients are potentially very severe, and impact directly on the quality of care offered by the health system [12]. Some studies inform about a direct relationship between burnout and patients’ mortality [13,14]. Others inform that burnout increases patients’ and families complaints to the institution [15]. Nurses can also present different symptoms such as insomnia, musculoskeletal pain, headache or institutions can also suffer nurses’ absenteeism [16].

Burnout levels may vary according to the unit where the nurses work [17,18,19,20], hence the need to analyse potential levels of burnout among workers in each professional category [21].

There is a wide research on nurse burnout syndrome in emergencies or critical care areas, in oncology services or in primary care coming from different countries and showing that nursing burnout is a global problem [10,17,18,19]. However, studies of burnout in medical area (MA) nurses are fewer and contradictory. The MA is defined as the physical space within an institution dedicated to health care or study, containing clinical hospitalisation units or services (such as cardiology, pneumology and haematology) presenting common characteristics. It is a large area, occupying most of the units in a hospital, and so it is here that the largest number of nurses with similar characteristics is liable to be affected by burnout. 

This study has the following main aims: to determine burnout levels; to identify the stage of burnout experienced by nurses working in the MA of hospitals in the Andalusian Health Service; to analyse the relationship between sociodemographic, occupational and personality variables and the occurrence of burnout syndrome in these professionals.

## 2. Material and Methods

### 2.1. Design

Quantitative, observational, cross-sectional, multicentre study.

#### 2.1.1. Sample/Participants

The sample population for this study was composed of 301 nurses working in the MA of 19 hospitals in Andalusia (southern Spain), in the following services: internal medicine, infectious disease, cardiology, pneumology, digestive medicine, neurology, nephrology, rheumatology, endocrinology, oncology, haematology, mental health and toxicology. The average age of the participants was 44.62 years (Standard deviation (SD) = 8.08), and 71.1% were female. All had higher education qualifications in nursing and were employed in their professional capacity as nurses. 

#### 2.1.2. Recruitment Process

Nurses working in the medical area for more than six months and who agreed to participate were included in the study. The questionnaires were given in a sealed envelope to all nurses in the 19 hospitals selected. A total of 400 questionnaires were distributed with the collaboration of the Spanish Nursing Union, and 324 envelopes were collected (81% response rate). Finally, *n* = 301 were completely filled out.

#### 2.1.3. Data Collection

Study data were compiled for the following sociodemographic and occupational variables. Sociodemographic data: sex (male–female), age (years), marital status (single, married or separated/divorced) and number of children (none, one, two, three or more). Occupational data: work routine (stable hours vs. shift work), on-call duties (yes–no), seniority in the workplace (months) and seniority in the profession (months).

Burnout syndrome was measured using the MBI [2] adapted for use with a Spanish population [22]. This instrument consists of 22 items, scored on a seven-point Likert response scale ranging from 0 (never) to 6 (every day). The MBI result is presented by reference to three dimensions: emotional exhaustion (nine items), depersonalisation (five items) and personal accomplishment (eight items). High burnout scores are defined according to previously-established cut-off points for a Spanish population, in each of the MBI dimensions: >24 for EE, >9 for D and <33 for PA. High scores for EE and D and low ones for PA are indicative of burnout. The following reliability coefficients (α) for the MBI scales were calculated: EE (α = 0.86), D (α = 0.61) and PA (α = 0.81).

The personality variables were measured using the Revised NEO Five Factor Inventory (NEO-FFI) Personality Inventory instrument [23], adapted for use with a Spanish population [24]. It is composed of five personality factors: neuroticism, agreeableness, conscientiousness, extraversion and openness to experience, spanning 60 items scored on a five-point Likert response scale, with twelve items for each dimension. The following reliability coefficients for the NEO-FFI dimensions were calculated: neuroticism (α = 0.78), agreeableness (α = 0.70), conscientiousness (α = 0.78), extraversion (α = 0.78) and openness (α = 0.69).

The Educational–Clinical Questionnaire: Anxiety and Depression (CECAD) [25] was used to measure symptoms of anxiety and depression, following the Diagnostic and Statistical Manual of Mental Disorders, Fifth Edition (DSM-V) criteria [26]. This questionnaire consists of 45 items scored on a five-point Likert response scale, with 20 items for the anxiety scale and 29 for the depression scale. The following reliability coefficients for the CECAD dimensions were calculated: anxiety (α = 0.92) and depression (α = 0.93).

Data for this quantitative, observational, cross-sectional and multicentre study were compiled by the research team in collaboration with the Spanish Nursing Union (SATSE), which provided the necessary information to MA nurses working in the Andalusian Health System. Participation was voluntary, individual and anonymous. The estimated time needed to complete the study questionnaire was 45 min. The data were collected during the second semester of 2017.

### 2.2. Ethical Considerations

The study was approved by the Ethics Committee of the University of Granada, and the ethical considerations of the Declaration of Helsinki [27] were complied with at all times. The data were processed in accordance with the provisions of Act 15/1999, of 13 December, on the Protection of Personal Data.

### 2.3. Data Analysis

Descriptive statistics—the mean, standard deviation, minimum and maximum values—were obtained for the quantitative variables, and frequencies and percentages for the qualitative ones. Student’s *t*-test for independent means was used to study the differences in the MBI dimensions according to the qualitative variables included in the study. Pearson’s correlation coefficients were obtained to study the linear relationship between the quantitative variables. Finally, backward elimination multiple linear regression was performed for each MBI dimension. All analyses were carried out using the SPSS 22.0 (IBM, Armonk, NY, USA) statistical package.

## 3. Results

### 3.1. Data, Burnout Levels and Estimated Prevalence

Table 1 shows the percentages and frequencies obtained for the qualitative variables included in the study. Among other notable findings, 71.1% of the nurses were female, 75.1% were married and 78.5% had at least one child. With respect to the occupational variables, 66.9% worked a fixed shift (morning, afternoon or night) and 89.8% did not have ‘on-call’ duties.

Table 2 shows the descriptive statistics obtained—mean, standard deviation, minimum and maximum—for the quantitative variables included in the study. The sociodemographic variable was age, the occupational ones were seniority in the profession and in the workplace, and the psychological ones were the personality dimensions of anxiety and depression, together with the three dimensions of burnout, EE, D and PA.

The following results were obtained for the three dimensions of burnout: 42.3% of respondents presented a low level of EE, 35.6% a moderate level and 22.1% a high level; 37.1% presented a low level of D, 39.8% a moderate level and 23.1% a high level; finally, 45.5% presented a low level of PA, 26.1% a moderate level and 28.4% a high level.

The Golembiewski model [5] was then used to classify the participants according to the phase of burnout experienced. Phases 6, 7 and 8 of this model represent a high level of burnout. In the present study, 39.8% (*n* = 109) of the respondents had high levels of burnout, by this measure (Table 3).

### 3.2. Explanatory Models and Factors Associated with Each Dimension of Burnout

In the next stage of our analysis, the linear correlations between the MBI scales, the NEO-FFI scales and the anxiety and depression scores were determined. The EE scale presented statistically significant correlations with the five dimensions of the NEO-FFI and with the CECAD anxiety and depression scores. Statistically significant relationships were found between depersonalisation and the NEO-FFI and CECAD items. Similarly significant correlations were found between personal accomplishment and the NEO-FFI and anxiety and depression items (Table 4).

Differences of the means were calculated for the MBI dimensions, with respect to sex, work shift, ‘on-call’ duties, marital status and children. In the depersonalisation scale, statistically significant differences were found between men and women (t_(297)_ = 3.210, *p* = 0.001, *d* = 0.41), with male respondents presenting higher values in this dimension.

Multiple linear regression models were obtained for each of the MBI dimensions. For EE, the variables ‘on-call’ duties (B = 5.385, *p* = 0.002), agreeableness (B = −0.334, *p* < 0.001) and depression (B = 0.402, *p* < 0.001) were statistically significant predictors. The goodness of fit of the model presented a value of r^2^ = 0.377. For D, the variables age (B = −0.099, *p* = 0.005), seniority in the workplace (B = 0.006, *p* = 0.021), agreeableness (B = −0.321, *p* < 0.001), conscientiousness (B = −0.105, *p* = 0.018) and depression (B = 0.099, *p* < 0.001) were predictors. 40.6% of the variance was explained by the model. Finally, for PA, the variables depression (B = −0.079, *p* = 0.016), agreeableness (B = 0.181, *p* = 0.015), conscientiousness (B = 0.483, *p* < 0.001), extraversion (B = 0.178, *p* = 0.007), seniority in the profession (B = 0.016, *p* = 0.001) and seniority in the workplace (B = −0.014, *p* = 0.002) were predictors. 39.5% of the variability was explained by the model (Table 5).

## 4. Discussion

This study was undertaken with three main aims. The first of these was to estimate burnout levels in a population of nurses working in the MA in hospitals in Andalusia (southern Spain). In this respect, 39.8% of the respondents presented high levels of burnout, a finding that is consistent with previous research in this field with a similar sample size [10], according to which the prevalence of this syndrome was 37.5%. Nevertheless, for all dimensions of the syndrome, many of the respondents presented high levels of burnout, which is in line with the literature from other countries [28,29,30]. In our study, the nurses were working in the MA, in hospital services or units of similar characteristics, such as cardiology, pneumology or neurology, where the nurse-patient ratio is higher than in other hospital areas (such as surgical, critical care or paediatrics), and where the regime of family visits is much more flexible. Each of these factors can promote burnout [31].

The burnout dimension most significantly affected was that of personal accomplishment. Among our respondents, 45.5% presented low levels of PA, which corroborated earlier studies from other countries [32,33,34,35]. On the other hand, some studies observed higher levels of PA in the medical area [36], or that the dimension most strongly affected is that of EE in this area [37,38,39,40] or that of D, although to a lesser extent [41]. The fact that the PA dimension was the most affected may reflect the existence of routine in the hospitalisation area of the hospital and the fact that nursing functions are divided by tasks. Thus, patient care is no longer individual and personalised, but is transformed into the repeated performance of standard tasks, which are equally applicable to all patients, according to the timetable and operations schedule of the unit. This situation provokes a sensation of a lack of fulfilment and professional development, impacting on nurses’ emotional health and on the care they provide, as the outcome of the dehumanisation experienced in many cases [42,43].

Our second study aim was to determine the phase of burnout experienced by the nurses working in the MA in hospitals in Andalusia. In this respect, the largest group of respondents (21.5% of the sample) presented high levels of burnout, as measured by the Golembiewski model [5], a finding that is in line with earlier results [29,44,45]. Although few previous studies have used the above model, in which different phases of burnout are distinguished, it provides many advantages, especially regarding classification and diagnosis, and therefore its use is recommended [10].

The third study goal was to analyse the relationship between sociodemographic, occupational and personality variables and the burnout experienced by nurses. In this respect, there were statistically significant differences in depersonalisation between male and female nurses, with the men being more severely affected. This finding is supported by other studies related to the medical area [10,29,46]. This difference could be related to the different coping styles of men and women, which predispose to or protect against burnout. In this respect, men tend towards avoidance and distancing, and hence present higher levels of D [37,47,48,49]. In fact, some authors have long argued that the D dimension is in fact a coping style [50,51].

Regarding the psychological variables, a significant relationship was found between anxiety and depression and all the dimensions of burnout, which highlights the magnitude of the problem and the importance of its prevention, in order to reduce or prevent mental health problems among this group of health care providers. Although these results corroborate those reported in other studies in the medical area from other countries [39,44,47,52,53], further research is needed to determine whether anxiety and depression develop before, during or as a cause of the syndrome.

On analysing each burnout dimension separately, we find that EE presents statistically significant correlations with neuroticism, extraversion, openness, agreeableness, conscientiousness, anxiety and depression. The variables ‘on-call’ duties, agreeableness and depression are predictors of EE. Clearly, the obligation to work night and weekend shifts predisposes MA nurses to EE [49,54,55,56,57], as does the existence of ‘on-call’ duties. Nurses with positive personality factors for emotional stability, such as agreeableness, presented lower EE levels, which is in line with previous findings [10]. Emotional and physical exhaustion provoke the apathy, indecisiveness and anhedonia that are symptomatic of depression, which would explain the correlation observed between these variables [58,59,60].

Statistically significant relationships were found between depersonalisation and neuroticism, extraversion, openness, agreeableness, conscientiousness, anxiety and depression. In the D dimension, the variables age, seniority in the workplace, agreeableness, conscientiousness and depression were all predictors of burnout. According to some authors, young nurses are more prone to D [28,33,61], although others argue that it is those who have been working longest in a given situation who present the highest levels of D [36,41,45]. The latter result is of great interest, as seniority in the workplace is often a moderating variable of burnout [10].

Finally, statistically significant correlations were found between PA and neuroticism, extraversion, openness, agreeableness, conscientiousness, anxiety and depression. In this dimension, the variables depression, agreeableness, conscientiousness, extraversion, seniority in the profession and seniority in the workplace were found to be predictors of burnout. The nurses who felt stronger feelings of PA were more effective and in control of their professional environment, with less stress and greater job satisfaction. All of these outcomes are favoured by greater age, experience and job stability [30,62]. Various personality traits can influence PA, since this feeling depends considerably on how self-efficacy is assessed, on the working conditions experienced, on the hostility or otherwise of the work environment, on relationships with patients [63,64]. Therefore, if emotional well-being is lacking, depression can develop, reducing the quality of care provided and hence patient safety [65,66].

The main limitation of this study is the relatively small sample size used to represent the large population of nurses who work in the medical area of hospitals. Nevertheless, the sample used is varied, and includes nurses working in different hospital services and cities throughout the region, which increases the representativeness of the sample.

Another question is the cross-sectional nature of the study design, which limits the possibilities for establishing associations of causality (to determine which factors may predispose nurses to suffer burnout), and so in future research it would be useful to conduct longitudinal studies, to broaden the research scope and to extend our analysis of the personality variables.

Finally, although all the personality variables initially considered have been analysed, others such as coping or resilience are also relevant and should be included in future studies of burnout. In addition, a more detailed consideration should be made of appropriate diagnostic tools for burnout syndrome, taking into account not only the Golembiewski phase model but also other methods based on physiological parameters, such as salivary biomarkers, which can reveal high levels of stress, as a further cause of burnout.

## 5. Implications for Clinical Practice

This paper describes a study conducted to determine the prevalence of burnout syndrome in almost 40% of the nurses working in the medical area of hospitals in Andalusia. The results obtained reflect high levels of burnout and very low levels of personal accomplishment, which is the dimension of personality most severely affected by the syndrome.

On the basis of these results, a profile can be constructed of the type of nurse most likely to present burnout syndrome. The creation of such profiles enhances our understanding of those who suffer from burnout, and of why some nurses are more vulnerable than others, despite working in the same service, performing the same duties, earning the same salary, working the same hours, etc.

We show that personality variables influence the likelihood of a nurse experiencing burnout, and that agreeableness, openness and extraversion are protective factors, while neuroticism and conscientiousness exacerbate the risk.

Our study results also highlight the undeniable correlation between burnout syndrome and anxiety and depression, highlighting the dangers of these mental health problems. Both have a negative impact on workers’ health, performance and productivity. Moreover, the anxiety and depression suffered by nurses can have negative consequences on the quality of care provided and hence on patients’ health. In summary, the large-scale reality of nurses who are anxious, depressed and suffering professional burnout, as revealed in our findings, is deeply unsatisfactory and a situation to be avoided in a hospital environment. 

To avoid this, there are some complementary therapies like yoga or mindfulness informing on the increase of subjective well-being, perceived stress, compassion, coping, resilience and reducing the levels of burnout [67,68,69,70]. Similar results have been obtained by physical practice and wellness courses for distress reduction and wellbeing improvement [71]. Finally, general self-efficacy and stress management can act as protective factors [72].

## 6. Conclusions

Burnout currently affects 39.8% of nurses working in the medical area of hospitals in the Andalusian Health Service. Measured on the eight-phase Golembiewski model of burnout severity, 21.5% of the nurses analysed present phase 6 or higher, i.e., they are experiencing high levels of burnout. The five personality variables considered, together with anxiety and depression, are significantly associated with the presence of all three dimensions of burnout syndrome in these nurses. Further research is needed to extend our understanding of the role of personality variables, in order to determine whether they are predictors of or protectors against burnout.

## Figures and Tables

**Table 1:** Descriptive data for the qualitative study variables.

Variable	%(*n*)	Variable	%(*n*)
Sex		Marital status	
Male	28.9(87)	Single	16.8(50)
Female	71.1(214)	Married	75.1(223)
Shift		Separated/Divorced	8.1(24)
Fixed	66.9(200)	Children	
Variable	33.1(99)	None	21.5(64)
On-call		One	19.1(57)
Yes	10.2(30)	Two	45(134)
No	89.8(264)	Three or more	14.4(43)

**Table 2:** Descriptive data for the quantitative study variables.

Variable	Mean (SD)	Min-Max
Age (*n* = 300)	44.62(8.08)	22–61
Seniority: Workplace (*n* = 289)	121.55(104.10)	1–456
Seniority: Profession (*n* = 300)	255.56(100.43)	12–488
NEO-FFI		
Neuroticism (*n* = 296)	27.05(7.38)	12–48
Extraversion (*n* = 299)	43.03(7.05)	20–60
Openness (*n* = 298)	39.16(6.74)	21–58
Agreeableness (*n* = 297)	45.65(6.14)	28–60
Conscientiousness (*n* = 298)	47.94(6.18)	30–60
CECAD		
Anxiety (*n* = 298)	35.65(10.86)	19–71
Depression (*n* = 298)	48.89(13.80)	26–91
MBI		
Emotional exhaustion (*n* = 298)	17.34(10.60)	0–54
Depersonalisation (*n* = 299)	6.10(5.05)	0–23
Personal accomplishment (*n*=299)	36.67(8.15)	14–48

SD = Standard deviation; CECAD = The Educational–Clinical Questionnaire: Anxiety and Depression; MBI = Maslach Burnout Inventory; NEO-FFI = Revised NEO Five Factor Inventory.

**Table 3:** Prevalence of burnout according to the stages of the Golembiewski model.

Phase	1	2	3	4	5	6	7	8
EE	L	L	L	L	H	H	H	H
D	L	H	L	H	L	H	L	H
PA	L	L	H	H	L	L	H	H
*n*	39	14	57	26	29	59	35	15
(%)	(14.2)	(5.1)	(20.8)	(9.5)	(10.6)	(21.5)	(12.8)	(5.5)

H = High; L = Low; EE = Emotional exhaustion; D = Depersonalisation; PA = Personal accomplishment.

**Table 4:** Correlation coefficients between psychological variables and burnout.

	EE	D	PA
NEO-FFI			
Neuroticism	0.505 **	0.392 **	−0.357 **
Extraversion	−0.361 **	−0.307 **	0.397 **
Openness	−0.174 **	−0.195 **	0.218 **
Agreeableness	−0.345 **	−0.520 **	0.417 **
Conscientiousness	−0.293 **	−0.362 **	0.524 **
CECAD			
Anxiety	0.514 **	0.397 **	−0.326 **
Depression	0.572 **	0.446 **	−0.372 **

EE = Emotional exhaustion; D = Depersonalisation; PA = Personal accomplishment; ** = *p* < 0.001.

**Table 5:** Multiple linear regression.

	B	Standard Error	Beta	*t*	*p*	95% CI
EE						
Intercept	12.494	4.835		2.584	0.010	2.977, 22.011
On-call	5.385	1.722	0.146	3.127	0.002	1.995, 8.774
Agreeableness	−0.334	0.085	−0.193	−3.917	0.001	−0.502, −0.166
Depression	0.402	0.038	0.521	10.614	0.001	0.328, 0.477
R^2^ = 0.377						
D						
Intercept	24.590	3.016		8.152	<0.000	18.651, 30.528
Age	−0.099	0.035	−0.160	−2.857	0.005	−0.167, −0.031
Seniority:workplace	0.006	0.003	0.129	2312	0.021	0.001, 0.012
Agreeableness	−0.321	0.044	−0.390	−7.326	<0.001	−0.408, −0.235
Conscientiousness	−0.105	0.044	−0.126	−2.388	0.018	−0.191, −0.018
Depression	0.099	0.019	0.268	5.173	<0.001	0.061, 0.137
R^2^ = 0.406						
PA						
Intercept	−0.889	5.061		−0.176	0.861	−10.852, 9.073
Depression	−0.079	0.033	−0.133	−2.418	0.016	−0.143, −0.015
Agreeableness	0.181	0.074	0.137	2.444	0.015	0.035, 0.326
Conscientiousness	0.483	0.072	0.360	6.697	<0.001	0.341, 0.625
Extraversion	0.178	0.066	0.153	2.702	0.007	0.048, 0.308
Seniority:profession	0.016	0.005	0.195	3.352	0.001	0.007, 0.025
Seniority:workplace	−0.014	0.005	−0.181	−3.097	0.002	−0.023, −0.005
R^2^ = 0.395						

B = Estimated parameter; CI = Confidence interval; EE = Emotional exhaustion; D = Depersonalisation; PA = Personal accomplishment; *t* = Student *t.*
